# Membrane association of the bacterial riboregulator Hfq and functional perspectives

**DOI:** 10.1038/s41598-017-11157-5

**Published:** 2017-09-06

**Authors:** Antoine Malabirade, Javier Morgado-Brajones, Sylvain Trépout, Frank Wien, Ileana Marquez, Jérôme Seguin, Sergio Marco, Marisela Velez, Véronique Arluison

**Affiliations:** 1 Laboratoire Léon Brillouin LLB, CEA, CNRS UMR12, Université Paris Saclay, CEA Saclay, 91191 Gif-sur-Yvette, France; 2 Instituto de Catálisis y Petroleoquímica, CSIC, c/Marie Curie, 2, Cantoblanco, E-28049 Madrid, Spain; 3 Institut Curie, Research Center, PSL Research University, Chemistry, Modelisation and Imaging for Biology (CMIB) Bât 110-112, Centre Universitaire, 91405 Orsay, France; 4 INSERM U 1196, CNRS UMR 9187, Université Paris Saclay, Université Paris-Sud, Bât 110-112, Centre Universitaire, Rue Henri Becquerel, 91405 Orsay, France; 5 DISCO Beamline, Synchrotron SOLEIL, 91192 Gif-sur-Yvette, France; 6 Institute for Integrative Biology of the Cell (I2BC), CEA, CNRS, Univ. Paris-Sud, Université Paris-Saclay, 91198 Gif-sur-Yvette, Cedex France; 7 Université Paris Diderot, 75013 Paris, France

## Abstract

Hfq is a bacterial RNA binding protein that carries out several roles in genetic expression regulation, mainly at the post-transcriptional level. Previous studies have shown its importance in growth and virulence of bacteria. Here, we provide the direct observation of its ability to interact with membranes. This was established by co-sedimentation assay, cryo-transmission electron (cryo-TEM) and atomic force (AFM) microscopies. Furthermore, our results suggest a role for its C-terminus amyloidogenic domain in membrane disruption. Precisely, AFM images of lipid bilayers in contact with Hfq C-terminus fibrils show the emergence of holes with a size dependent on the time of interaction. Cryo-TEM observations also show that liposomes are in contact with clusters of fibrils, with occasional deformation of the vesicles and afterward the apparition of a multitude of tiny vesicles in the proximity of the fibrils, suggesting peptide-induced breakage of the liposomes. Finally, circular dichroism spectroscopy demonstrated a change in the secondary structure of Hfq C-terminus upon interaction with liposomes. Altogether, these results show an unexpected property of Hfq and suggest a possible new role for the protein, exporting sRNA outside of the bacterial cell.

## Introduction

The bacterial Hfq protein was identified in *Escherichia coli* as an abundant RNA-binding protein about fifty years ago^1^. Today Hfq is recognized as the core component of a global post-transcriptional network that facilitates the imperfect base-pairing of small regulatory noncoding RNA (sRNA) with trans-encoded mRNA targets^2, 3^. Consequently, Hfq induces the repression or activation of translation, with important related consequences for RNA stability^4^. This mode of regulation is of primary importance for the adaptation of bacteria to the changing environment, and thus for the control of cell division and for the virulence of pathogenic species^5, 6^.

The ability of Hfq to interact with DNA has also been demonstrated and it has been described as one of the *E*. *coli* nucleoid associated proteins (NAP) *in vivo* ^7, 8^. Nevertheless, the fraction of Hfq within the nucleoid represents only 10–20% of the total protein concentration, while approximately 50% of the protein is found in close proximity of the membrane^9, 10^. This makes sense as the majority of Hfq-regulated sRNA targets encode membrane proteins^11^.

Regarding its molecular structure, *E*. *coli* Hfq forms an Sm-fold in its N-terminal region (NTR, 2/3 of the 102 amino acid residues protein)^12, 13^. This fold consists of a five-stranded antiparallel β-sheet capped by an N-terminal α-helix. The β-sheets from six monomers interact with each other to assemble in a toroidal structure with two different surfaces^14^. It appears that the distal face and the edge of the protein are involved in both DNA and RNA binding, while the proximal face (on which the α-helix is exposed) seems to be involved in RNA binding only^15, 16^. Besides its Sm-like domain, other regions of Hfq such as the C-terminal region (CTR) also play a role in nucleic acid recognition and binding^17–19^. The Hfq three-dimensional structures of various bacteria have been resolved^20–24^, but until now all lack the CTR, so the way this Hfq region folds remains enigmatic. Structurally, the CTR seems to extend outside the Sm-core and appears to be intrinsically disordered^25, 26^. This domain is also presumably important for protein stability^25, 27^. Recently, it has been shown that the CTR region of *E*. *coli* Hfq contains an amyloid sequence^28^. This allows the Hfq protein to self-assemble, a feature common to Hfq and Sm proteins^29, 30^.

In this work, we focus our attention on a new unexplored feature of Hfq, namely its ability to bind to membranes. This finding opens new perspectives in Hfq-dependent riboregulation that will be discussed herein.

## Results

### Hfq CTR and NTR domains both interact with lipid bilayers

**Figure 1:**
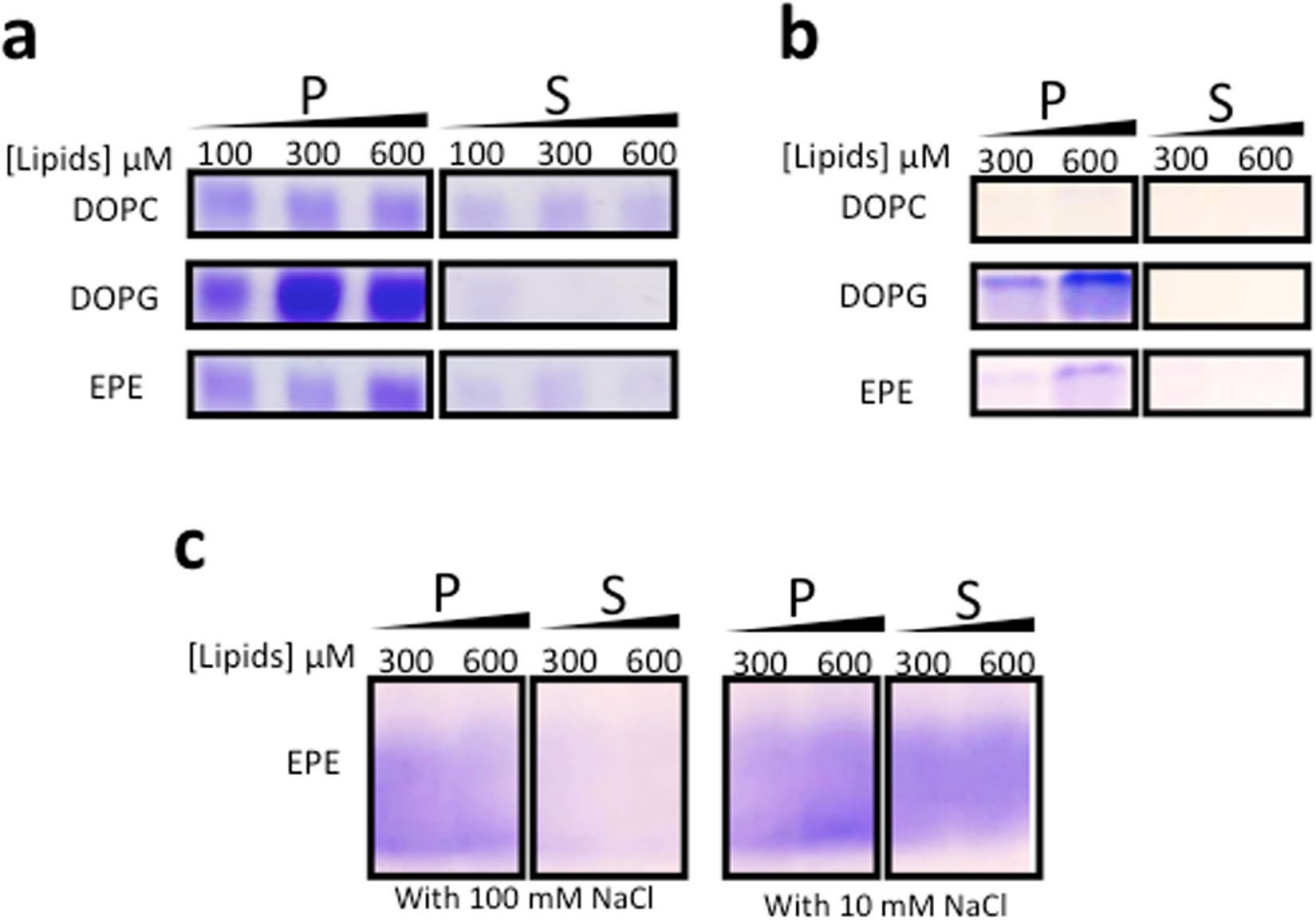
Co-sedimentation assay of Hfq with select lipids. SUVs in SUV buffer (10 mM Tris-HCl pH 7.5, 100 mM NaCl) were incubated with 3 µM Hfq-WT/Hfq-NTR_65_ or 126 µM Hfq-CTR_38_. (**a**) Wild-type Hfq sediments with a preference for polar lipids. (**b**) The same qualitative result is obtained with truncated Hfq-NTR_65_. (**c**) Hfq-CTR_38_ peptides also bind SUVs and the interaction is promoted by high ionic strength. P, Pellet fraction; S, Supernatant fraction. Gels were different for Hfq-WT, Hfq-NTR_65_ and Hfq-CTR_38_ peptides, but for each condition protein analysis was made on the same gel. Note that as previously described, Hfq-WT hexamers are only partially denatured by SDS-PAGE and migrate mostly as hexamers^27, 60, 61^, while truncated Hfq-NTR_65_ is less stable and can be dissociated in the PAGE^25, 27^ (Fig. S6). Protein concentrations were chosen in order to have similar mass concentrations.

In order to have a broad view of the ability of Hfq to interact with lipid bilayers, several lipids were first tested for potential interactions by co-sedimentation assays. In all cases, we observed that the protein alone at the concentration specified did not sediment in the conditions used. Results are presented in Fig. 1. The wild-type Hfq hexamer has a pronounced affinity for dioleoylphosphatidylglycerol (DOPG) polar lipids, which are present in the natural inner membrane of *E*. *coli* and therefore in the *E*. *coli* polar extract (EPE) mixture, partially explaining the good affinity for the EPE small unilamellar vesicles (SUVs) (Fig. 1a). Only a small interaction is observed with dioleoylphosphatidylcholine (DOPC), an example of less polar lipids. This result is however not surprising considering the highly hydrophilic and positively charged *E*. *coli* Hfq surface. A comparable behavior is obtained with truncated Hfq-NTR_65_ missing the Hfq CTR tail (Fig. 1b).

We also investigated the Hfq-CTR_38_ propensity to interact with SUVs, knowing its tendency to aggregate into amyloid fibers^28^ and the natural property of such a domain to interact with membranes^31^. Results with the 38 amino-acid peptide corresponding to Hfq CTR tail are presented in Fig. 1c, showing a significant binding of CTR peptide to EPE lipids. Note that this interaction is promoted by the presence of NaCl, and the opposite is observed for the NTR domain alone, thus suggesting a different mode of interaction.

### Clusters of Hfq are in direct contact with lipid bilayers

**Figure 2:**
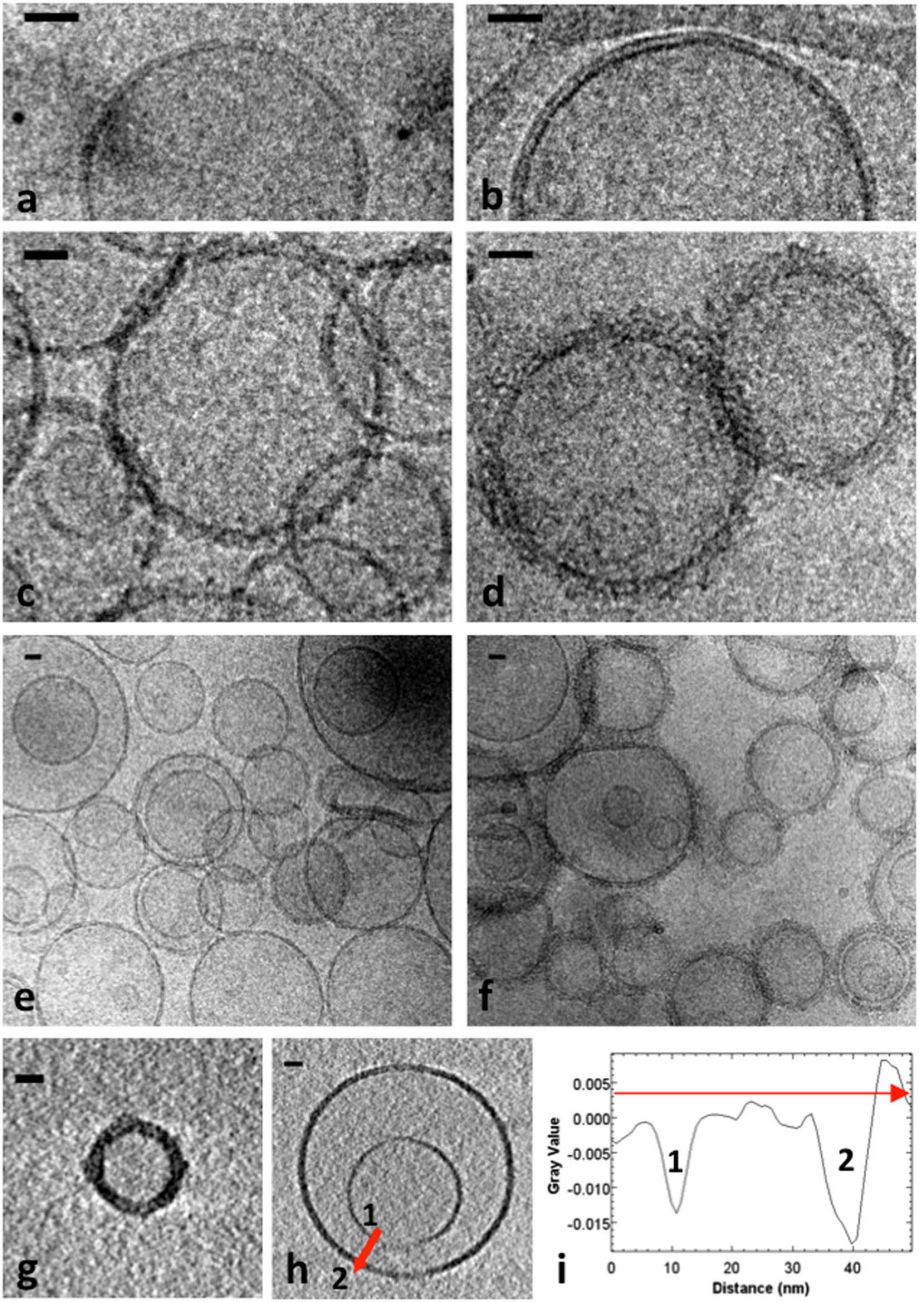
Cryo-microscopy images and tomography of wild-type Hfq bound to SUVs. (**a**) Liposomes composed of 100% EPE lipids show a clean bilayer. (**b**) Same as (**a**) but with DOPG lipids. (**c**) EPE liposomes incubated with 10 µM Hfq. (**d**) DOPG liposomes incubated with 10 µM Hfq. (**e**) and (**f**) Same as (**c**) and (**d**) with lower magnification, respectively. (**g**) Cryo-tomography of Hfq and DOPG SUVs showing a bilayer completely covered by proteins, which eventually form aggregates visible on the surface. (**h**) Tomography of double DOPG liposomes showing that only the outer membrane (2) is decorated by Hfq, and is thicker. The inner membrane (1) has an undecorated bilayer. ***i***. Grey intensity profile is displayed according to the red arrow on image (**h**), showing the thickness difference between liposome 1 and 2. Scale bars: 20 nm.

As seen by Cryo-electron microscopy (cryo-TEM), SUVs formed from EPE or DOPG lipids are round-shaped with a mean diameter ~ 120 nm (Fig. 2a and b). The membrane bilayer with the expected thickness of 4.1 ± 0.3 nm is clearly observed (in agreement with previous reports^32^). Liposomes were sparsely distributed over the grid and are mostly found isolated. Mixing SUVs with full-length WT Hfq induces SUVs clustering. The liposomes are decorated with proteins, leading to a significant increase of membrane thickness: 7.6 ± 1.0 nm or 8.2 ± 1.2 nm for EPE or DOPG liposomes, respectively (Fig. 2c–f). A subtraction between the respective membrane thickness of uncoated and coated liposomes gives a layer of protein measuring approximately 3–3.5 nm for EPE. As Hfq hexamer measures about 3 nm in height, this suggests that hexamers are lying horizontally on the membrane. Note that the protein layer is not completely flat, reflecting a complex arrangement, and that the bilayer is sometimes no longer visible, suggesting that Hfq may sometimes affect the membrane surface. Furthermore, proteins cover almost all the DOPG liposomes, showing some thicker stacks, whereas the EPE SUVs are not always decorated (Fig. 2e). This is consistent with the apparent higher affinity for DOPG observed by co-sedimentation. In order to evaluate the global shape and protein coating of SUVs, we also performed cryo-tomography. 3D reconstruction gives very thick liposomes, normally shaped and fully decorated by proteins, in accordance with TEM results (Fig. 2g). Occasionally, a small SUV is trapped inside a larger one coated by Hfq (Fig. 2h), allowing clear observation of the different thicknesses; measurement of the membranes thickness along the red arrow gives approximately 3.87 nm for the inner liposome^32^ and 6.82 nm for the outer one, in accordance with measurements obtained on projections (Fig. 2i).

**Figure 3:**
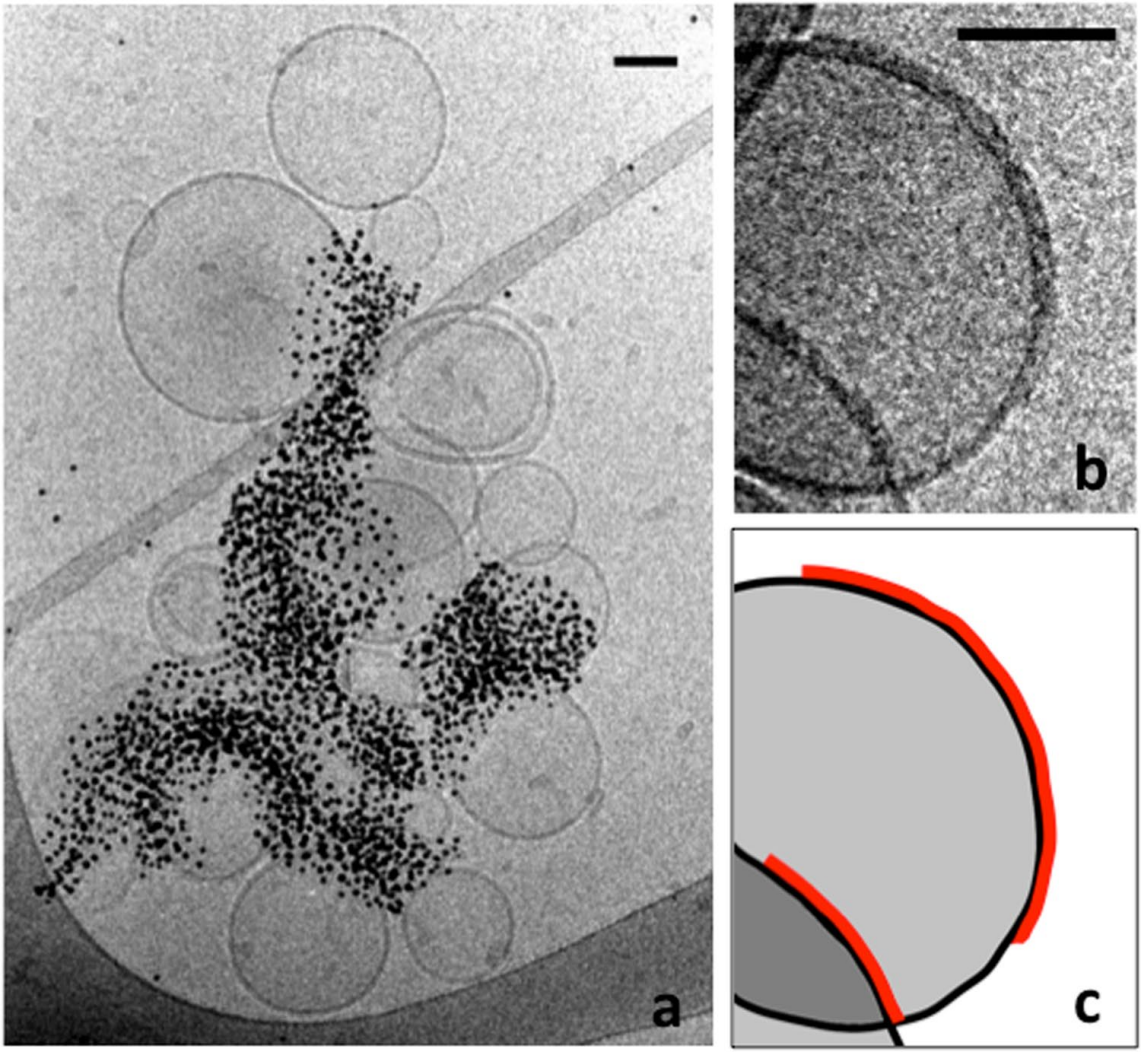
Cryo-microscopy images of Hfq bound to SUVs. (**a**) EPE SUVs incubated with 3 µM His-tagged Hfq. 4 nM Ni-NTA-NanoGold beads were added a few seconds before freezing. (**b**) EPE SUVs incubated with 10 µM wild-type Hfq. ***c***. Schematic drawing underlining membranes (black) and protein layers (red). Note the formation of Hfq clusters at the liposome interface, suggesting a cooperative binding of the protein to membranes. Scale bars: 50 nm.

To prove that Hfq promotes liposome-liposome contacts, we then used C-terminal His-tagged Hfq in the presence of Ni^2+^-functionalized gold beads, to denote the position of the proteins (Fig. 3a). Note that in this condition, the His-tagged protein concentration is three times lower than the wild-type Hfq concentration used in Fig. 2. Control experiments showing beads and liposomes without protein (Fig. S1a) and beads weakly bound to the natural histidines of the wild-type protein (Fig. S1b) are provided as supplementary data. As seen, gold beads are not randomly coating liposomes but clustered, suggesting that proteins are organized in clusters (possibly fibers) at the liposome-liposome interfaces. This observation is consistent with the partial decoration of liposomes, indicating that Hfq protein tends to bind preferentially to protein-lipid clusters already formed (Fig. 3b and highlight in Fig. 3c). This observation may suggest that Hfq binding to membranes is cooperative.

**Figure 4:**
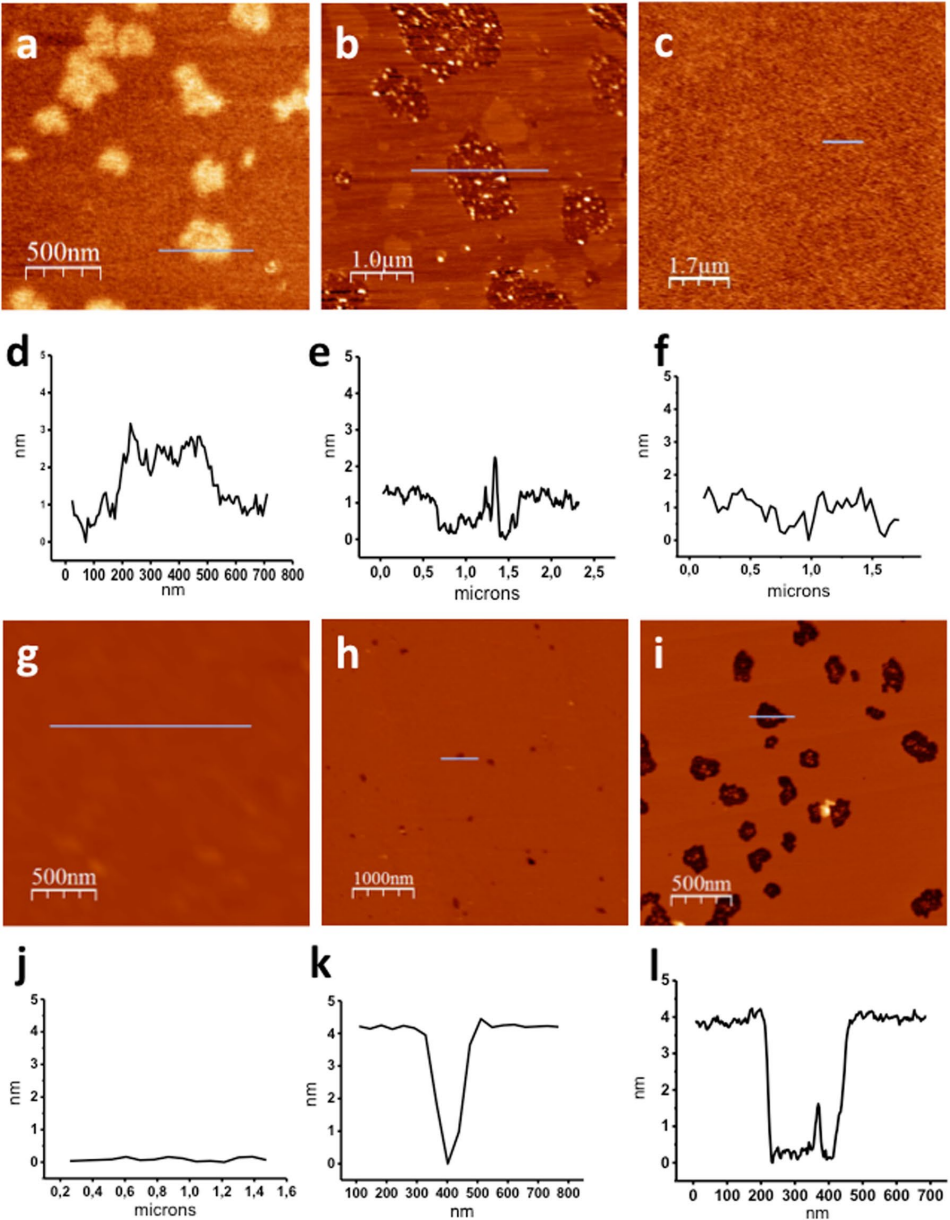
AFM images of wild-type Hfq at 2 µM (*a*,*b*,*d*,*e*), Hfq-NTR_65_ at 2 µM (*c*,*f*), or Hfq-CTR_11_ at 176 µM (*g-l*) in solution on an EPE supported bilayer. (**a**) After 5 min incubation, Hfq binds on the top and forms groups of several hexamers. (**b**) After 1 h incubation, the bilayer is affected and 1–2 nm deep holes appear, containing undetermined material inside. (**c**) After 1 h incubation, no trace of Hfq-NTR_65_ is visible and the membrane is clean. *g*. Clean bilayer before adding Hfq-CTR_11_. *h*. After 30 min incubation, small 4 nm deep holes start to form. *i*. After 1 h incubation, holes have grown to several hundreds of nm wide, containing undetermined material inside. *d*, *e*, *f*, *j*, *k* and *l*, height profiles of grey lines on images *a*, *b*, *c*, *g*, *h* and *i*, respectively. Protein concentrations were chosen in order to have similar mass concentrations.

In order to confirm this result, we then used atomic force microscopy (AFM) in solution to observe Hfq behavior on supported lipid bilayers. As expected, after a short incubation time on an EPE bilayer, proteins are distributed over the surface in small aggregates, compatible with hexamers regrouped in clusters (Fig. 4a). The aggregates have an average height of 2.7 ± 0.2 nm, obtained from measuring the profile of 20 different aggregates (Fig. S2). These are lower than previous cryo-TEM measurements, but membrane curvature is different and the measurement is still compatible with a horizontal-lying torus that could slightly enter the first lipid layer.

### Full length Hfq affects the bilayer organization, but not HfqNTR_65_

Surprisingly, after longer incubation times, the membrane exhibits a drastic reorganization. Lipid domains appear, which is comprehensible when a positively charged protein interacts with anionic phospholipids present in the EPE^33^. However, we also observed the presence of numerous unexpected holes with a diameter around 100 nm and 1–2 nm in depth (Fig. 4b). As the membrane treated in the same condition in the absence of Hfq does not present such a reorganization on the same timescale, this indicates that Hfq affects the membrane integrity. Note that some undetermined material is still present inside the deep holes, which does not correspond to the ~4 nm thickness of the bilayer (Fig. 4e). We assume this could be Hfq or Hfq-lipids aggregates resulting from the membrane reorganization.

We then performed the same set of experiments with Hfq-NTR_65_. Cryo-TEM revealed that, to a lower extent than full length Hfq, Hfq-NTR_65_ was able to regroup liposomes, but no visible protein decoration is observed on the surface (Fig. S3). Furthermore, AFM with Hfq-NTR_65_ gives different results than with Hfq-WT, closer to a normal membrane (Fig. 4c,f). The bilayer is rougher than before being incubated with the protein, but does not undergo reorganization and holes formation even after a long incubation time. It suggests that the truncated protein adsorbs weakly on the membrane and is easily washed away when rinsing with buffer before imaging, or pushed by the AFM tip.

In order to understand which part of the protein is involved in the hole-formation observed with the full-length protein, we then investigated the effects of the amyloidogenic Hfq-CTR, which is also able to interact with EPE SUVs (Fig. 1c).

### Identification of the amyloid region in Hfq-CTR

First, the amyloid region within CTR domain of Hfq was identified precisely. To that end, various peptides corresponding to overlapping sequences in the CTR were synthetized according to Waltz algorithm predictions^34^ (see Table S1 for sequences). Self-assembly of these peptides was tested by cryo-TEM and positive results are summarized in Fig. S4. This allowed us to determine that a short sequence of 11 amino acids residues is responsible for the self-assembly, precisely the region SAQNTSAQQDS. We also confirmed by FTIR spectroscopy^35^ that this small peptide self-assembles into amyloid fibrils. This 11 amino-acid residues peptide will be referred to as Hfq-CTR_11_.

### Hfq-CTR_11_ amyloid fibers trigger liposome breaking and membrane disruption

**Figure 5:**
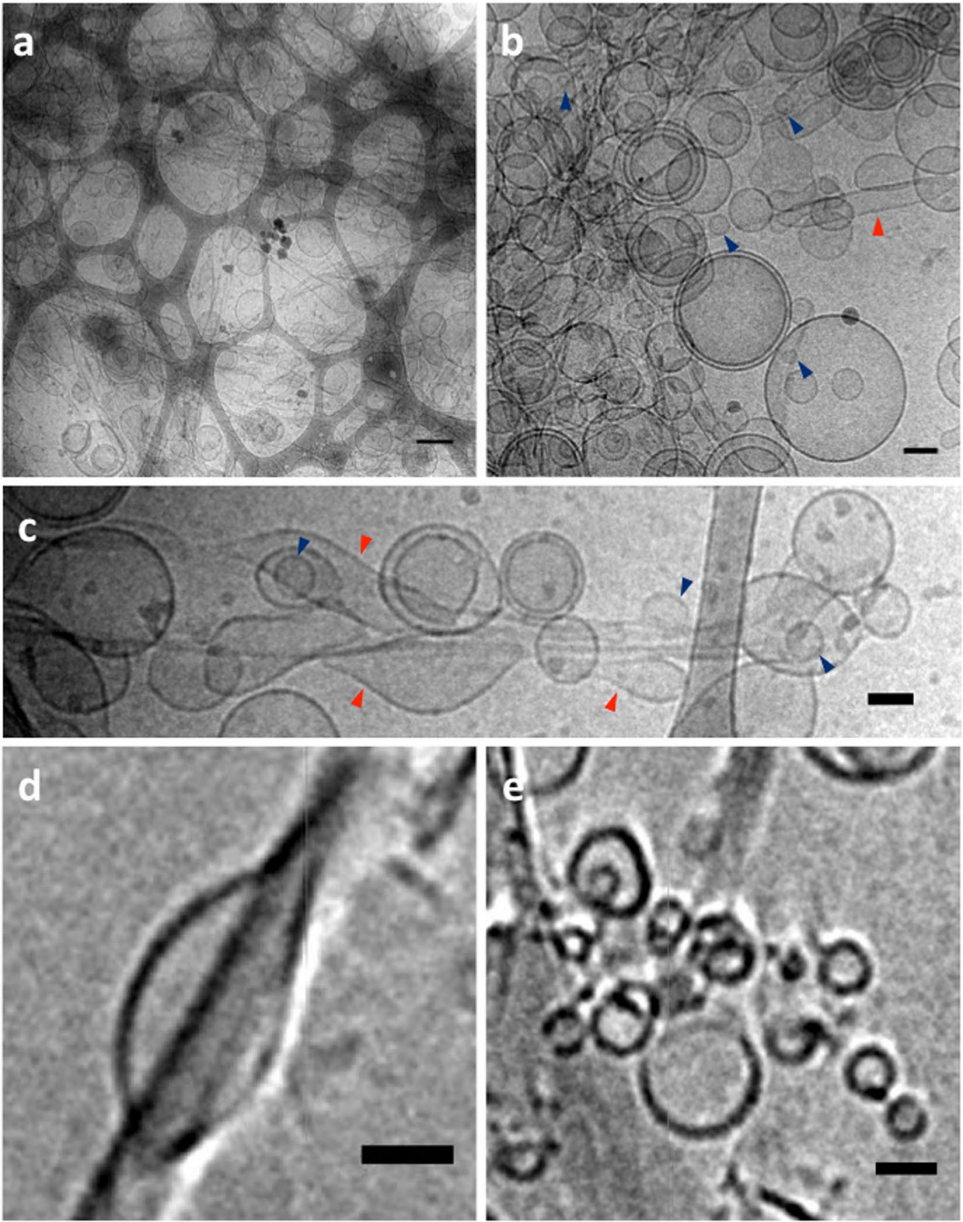
Cryo-TEM images and tomography of Hfq-CTR11 incubated with EPE SUVs. (**a**) Hfq-CTR_11_ peptides aggregate to form amyloid fibrils and regroup large clusters of liposomes. (**b**) A high proportion of small liposomes of 10–30 nm (blue arrows) are present, possibly coming from SUVs disruption by Hfq-CTR_11_.(**c**) Multiple contacts between liposomes and fibers create SUV deformations (red arrows). (**d**) Liposome deformation as seen by cryo-tomography. (**e**) Small liposomes (10–30 nm) as seen by cryo-tomography. Scale bars: 200 nm for (**a**), 50 nm for (**b**,**c**) and 20 nm for (**d**,**e**).

The same experiments carried out previously with the native protein were repeated with Hfq-CTR_11._ In this case, fibrils can be clearly observed with Cryo-TEM (peptide concentration 440 µM, *i*.*e*. 0.5 g.L^−1^) (Fig. 5). Stacks of fibrils decorated with liposomes form along a specific direction, different for each cluster (Fig. 5a). Liposome deformations at the liposome-fibril contact regions are frequent (red arrows Fig. 5b and c). Furthermore, many small liposomes of 10–30 nm appear (blue arrows Fig. 5), resulting from the rupture of larger SUVs. Note that, although the cryo-TEM indicates an interaction between fibrils and liposomes, it is not possible to determine from individual projections whether overlap occurs in the same plane of the sample. Therefore, cryo-electron tomography has been used to reconstruct the 3D structure of the interaction between fibrils and liposomes (Fig. 5d and e). Tomography images directly confirm contacts between the two components. Furthermore, analysis of the liposome morphology also demonstrates that some of the liposomes are deformed by fibrils (Fig. 5d) and small 10–30 nm liposomes are also observed (Fig. 5e).

Then, the effect of Hfq-CTR_11_ on EPE mica-supported lipid membranes was observed by AFM. Before protein incubation, they presented a roughness under 1 nm (Fig. 4g and j). After addition of Hfq-CTR_11_, small holes clearly appear (Fig. 4h) that grow with incubation time (Fig. 4i). The measured depth of these holes is 4 nm, which means that unlike the native protein, the Hfq-CTR_11_ peptide removes the whole bilayer (Fig. 4k and l), a result compatible with the SUV deformation and splitting observed in TEM. Again, we assume that protein/lipid aggregates are likely present in the holes formed (Fig. 4i). But in this case no aggregated protein is found adsorbed on the intact membrane area.

### Interaction with membrane induces a conformational change in Hfq CTR

**Figure 6:**
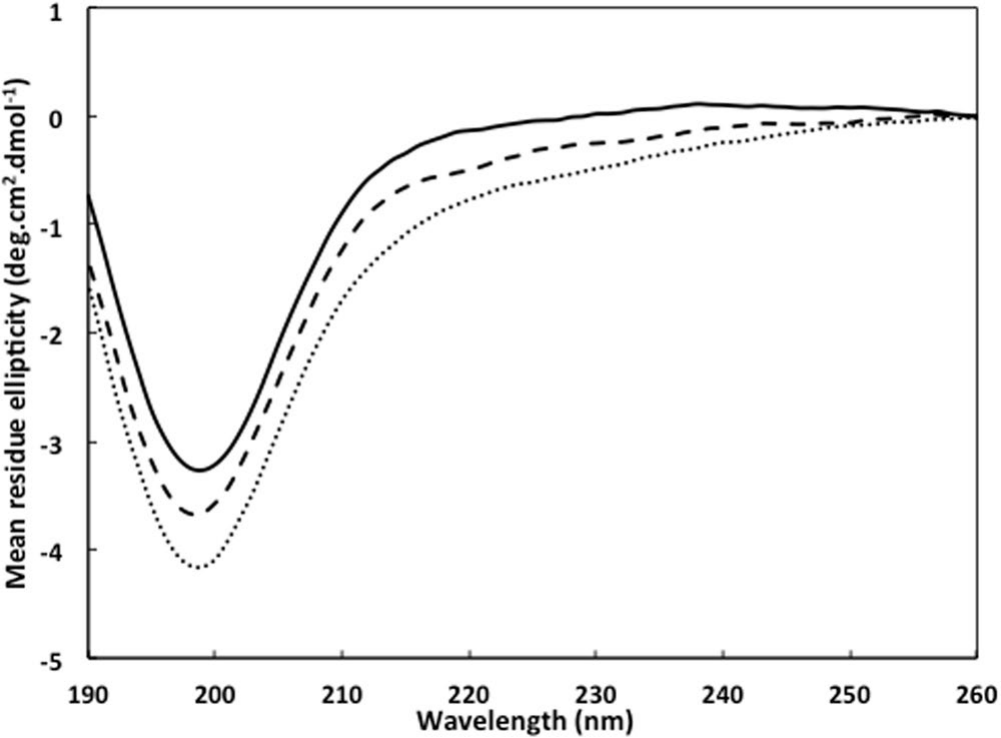
SRCD spectra of the Hfq-CTR_38_ peptide interacting with liposomes. Hfq-CTR_38_ alone (solid line), with EPE SUVs (dashed line) or with DOPG SUVs (dotted line). The respective contribution of buffer (10 mM sodium phosphate and 20 mM NaCl) and/or lipids were subtracted.

Finally, to assess how the Hfq-CTR is interacting with SUVs, we performed Synchrotron Radiation Circular Dichroism (SRCD). The SRCD spectra were obtained for freshly dissolved Hfq-CTR_38_ in solution and Hfq-CTR_38_ incubated with SUVs (Fig. 6). The respective secondary structure compositions were determined using Bestsel and are reported in Table 1. As expected from our previous observations^28^, the peptide alone is partially unstructured or folded in β−sheets (38%). When incubated with EPE liposomes, the percentage of β−sheets decreases to 33%, while when incubated with DOPG liposomes, the percentage of β−sheets is reduced to 28% with a small increase of turns and apparition of 5.6% α-helices (classified as “distorted helix” category according to Bestsel). In the later case, it is likely that only a fraction of the peptide is interacting the membrane and that more than 5–6% helices is formed in the population bound to membrane. Note that the helices formed seem to be unstable without the interaction of the peptides with the polar phosphoglycerol lipid heads.

**Table 1:** SRCD spectra analysis of Hfq-CTR_38_ in the absence and in the presence of liposomes. Analysis was performed using the Bestsel software. The results are given as percentage.

Secondary structure (%)	Hfq-CTR_38_	Hfq-CTR_38_ + EPE	Hfq-CTR_38_ + DOPG
α helix	0	0	5.6
Turn	14	15.8	17
Antiparallel β sheets	38.1	32.7	27.9
Others	47.9	51.4	49.4

## Discussion

Our results demonstrate that Hfq has a propensity to interact with membranes. Importantly, the native protein used for all experiments here was exempt of post-translational modification such as lipid addition. This has been confirmed by mass spectrometry analysis (Fig. S5). Thus, such a modification is not needed for interaction of the protein with a bilayer membrane *in vitro*, even if the existence of this modification has been reported previously^36^.

For Hfq-membrane interaction, two distinct actions may be described. First, a relatively weak, reversible binding with the torus lying on the membrane is observed. The affinity of the native protein for polar lipids suggests that binding is mainly driven by hydrogen bonding and electrostatic interactions. The importance of weak electrostatic interactions between basic residues and acidic lipids could be considered as a mean for proteins to be transiently released in the cytoplasm^37^ and could explain why Hfq was not identified in the membrane fraction in large-scale proteomic studies^38^.

The self-assembly of Hfq through its C-terminal tails at the bilayer surface also provides a possible explanation for the superstructures observed previously *in vivo* in close proximity of the inner membrane^10, 28, 39^. The C-terminal tails strengthen the protein-lipid interaction. As membrane association is lost when this CTR is deleted^28^, we assumed this part of the protein was essential for the interaction with membrane *in vivo*. Our present results with Hfq CTR region confirm this assumption. This result is also in agreement with previous works on the ability of fibrils including amyloid fibrils to interact with membranes, also in the case of peptide-forming fibers^31, 40, 41^.

The second action we have observed is a drastic change in the membrane organization with longer incubation times. This rearrangement is induced by the C-terminal amyloidogenic portion of the protein. AFM measurements suggest that the full-length protein remove one monolayer of the membrane, in agreement with the recent work of Pyne *et al*.^42^. Taking into account that monolayer poration has already been reported for other proteins^42–44^, one possibility is that similarly to Tilamin, Hfq-induced monolayer-pores may cause local membrane rupture^42^. Nevertheless, in the case of Tilamin, monolayer poration induces progressive removal of the outer membranes and bacterial death^42^. More likely in the case of Hfq, the protein induces transient pores *in vivo* and not membrane disintegration. We also confirm here the ability of amyloid regions to disrupt and distort membranes^45^.

Finally, the secondary structural change of Hfq-CTR_38_ observed by SRCD suggests that the amyloid-like region could be partially converted into a α-helix, possibly a trans-membrane helix as reported earlier for other amyloids^46^. Another possibility would be that an amphipathic helix lying horizontally in the acidic phosphate-heads layer forms, such as that observed for RNAse E or RNAse II^47, 48^. This would be in agreement with the flexibility of Hfq CTR and with its composition of both basic/polar (H, Q, N, T) and non-polar (A, Y) amino acid residues. Alternatively, a tilted helix may also form as in the case of Tilamin, resulting in its insertion in the monolayer^42^. These possibilities however need to be investigated further, for instance using orientated circular dichroism (OCD) to observe the orientation of the helix inside the membrane^49^.

In conclusion, the key finding of this work is that Hfq is able to affect biological membranes, as shown in AFM and cryo-TEM. This finding may support a possible mechanism by which Hfq could contribute to the export of RNA outside the bacteria or in the periplasmic space. To date, known exported molecules for quorum sensing were limited to small molecules. Nevertheless bacterial RNAs have been identified extracellularly^50^. Among those RNAs, small noncoding RNA are found. Here we propose a new possible role for Hfq in sRNA export, with important consequences for bacterial communication.

## Materials and Methods

### Protein expression and purification

Wild type *Escherichia coli* Hfq was purified as described previously^39^. Hfq-NTR_65_ (residues 1–65) was purified from over expressing BL21(DE3)∆hfq/pLATE11-hfqntr_65_ cells. The pLATE11-hfqntr_65_ expression vector was constructed according to the manufacturer protocol (Thermofisher). As the his-tag potentially modifies Hfq properties^51^, a sequence encoding the Tobacco Etch Virus (TEV) protease site (ENLYFQG) was inserted between the N-terminal His-tag and Hfq sequence. For the purification of Hfq-NTR_65_, cells from post-induction cultures (IPTG 1 mM, 3 h induction) were resuspended on ice in 20 mM Tris HCl pH 7.5 containing 0.5 M NaCl, 10% (v/v) glycerol and a protease inhibitor cocktail (Sigma). The cells were lysed by sonication and the lysate was cleared by centrifugation at 15,000 g for 30 min. DNase I (40 g.L^−1^) and RNase A (30 g.L^−1^) were added to the cleared lysate at room temperature. The solution was then applied to a His-trap column (GE Healthcare). The resin was washed with 20 mM Tris HCl pH 7.5 containing 0.3 M NaCl and 20 mM imidazole; the protein was eluted with a gradient of imidazole (20–500 mM) in the same buffer. The eluted protein was dialyzed in the appropriate buffer and TEV digestion was carried out according to the manufacturer’s instructions (Thermofisher). After digestion, Hfq-NTR_65_ was subsequently purified with a HiTrap SP HP 1 mL column (GE Healthcare). Before injection on the cation exchange column, Hfq-NTR_65_ was diluted five times in equilibration buffer (50 mM HEPES pH 8) to reduce salt concentration. The resin was equilibrated with the same buffer and the protein was eluted with a gradient of NaCl (0–1 M). The cleaved His-tag, non-digested Hfq-NTR_65_ and other remaining contaminants are eliminated with this cation exchange column.

Hfq C-terminus peptides were obtained from Proteogenix (France). Fibrils of synthetic peptides were prepared from dissolved peptide in deionized water at 0.5 g.L^−1^. Fibrils were usually observed after one week. Note that kinetics of assembly can be greatly affected by sample batch-to-batch variability. Several factors contribute to this variability, including the presence of salts in batches and the presence of various amounts of pre-formed aggregates in samples.

### Preparation of small unilamellar vesicles (SUVs)

Lipids purchased from Avanti Polar Lipids (Alabaster, AL, USA) were dissolved at 10 mg.mL^−1^ in chloroform/methanol 1:1 (v/v) on ice. The lipids used were *E. coli* Polar Extract (abbreviated EPE, ref 100600P), 1,2-dioleoyl-sn-glycero-3-phosphocholine (abbreviated DOPC, ref 850375) and 1, 2-dioleoyl-sn-glycero-3-phospho-(1′-rac-glycerol) (abbreviated DOPG, ref 840475). The solvent was slowly evaporated with N_2_(g), forming a lipid film that was then dried for 30 min under N_2_ flow. Lipids were hydrated in SUV buffer (10 mM Tris pH 7.5 containing 100 mM NaCl). Vesicles formed during slow stirring at room temperature over 30 min. The solution was then passed 31 times through a 0.2 µm polycarbonate filter (Avanti Mini Extruder) to obtain SUVs. Samples were kept at 4 °C and used for subsequent experiments within one week. For liposomes used in CD experiments, the protocol was identical but Tris-HCl was replaced by phosphate buffer (10 mM sodium phosphate buffer pH 7.5 with 100 mM NaCl) and using 0.1 µm polycarbonate filter for extrusion.

### Co-sedimentation assay

Gradual concentrations of SUVs (100, 300 and 600 µM lipids) were mixed with proteins in SUV buffer with or without NaCl and left 20 min at 22 °C before being ultracentrifuged 1 h at 100 000 g at the same temperature in a Beckman TL-100 ultracentrifuge with a Beckman TLA100 rotor. 10% of total sample volume were recovered from the supernatants and pellets which were then were loaded on polyacrylamide gels for SDS-PAGE. Gels were stained with R-250 or G-250 Coomassie Brilliant Blue.

### AFM imaging in solution

SUV suspension diluted at 0.4 g.L^−1^ in SUV buffer supplemented with 2 mM CaCl_2_ was incubated on a freshly-cleaved mica surface for one hour at 37 °C. The sample was then rinsed ten times with SUV buffer to remove excess SUVs. Supported bilayers were checked by AFM to confirm the extent of surface coverage and the quality of the lipid bilayer on the mica before protein incubation.

The proteins were added to the surface at a given concentration at room temperature for various times (from 5 min to several hours). Incubations were done in a sealed box to avoid evaporation. The sample was then rinsed ten times to remove unbound proteins and imaged in the same buffer. Protein concentrations were 2 µM for both native Hfq and Hfq-NTR_65_, and 176 µM for Hfq-CTR_11_ peptide (1136 g/mol). Protein concentrations were chosen in order to have similar mass concentrations.

In this work, commercial Olympus rectangular silicon nitride cantilevers (RC800PSA) and two different commercial AFMs were used: an Agilent technologies 5500 microscope (Santa Clara, CA, USA) operated in the tapping mode at a resonance frequency of 15 kHz and 0.73 N.m-1 spring constant cantilever and a Nanotec microscope (Madrid, Spain) operated in the jumping mode with an applied force <100 pN^52^. After the sample was installed on the AFM stage, about 30 min were required for the system to reach thermal equilibrium before commencing the AFM scans. We imaged 5 to 10 positions at different resolutions to ensure consistency among the observations.

Before obtaining the height profiles from the AFM images, the tilt of the surface and the line to line noise were removed using the plane and flattening filters provided in the WSxM free software (www.wsxmsolutions.com)^53^. The height profiles were all obtained from line scans perpendicular to the slow scanning direction.

### Cryo-Electron microscopy (cryo-TEM)

SUVs in SUV buffer (0.2 g.L^−1^ final concentration) and protein in the same buffer were mixed and left at room temperature for 20 min. When necessary, 5 nm Ni-NTA-NanoGold beads (NanoProbe, Yaphank, NY) were added 2 minutes before freezing at a final concentration of 4 nM in order to label His-tagged proteins. The high clustering observed in cryo-TEM images originates from the fact that Ni-NTA gold beads possess multiple Ni-NTA moieties that can bind several liposomes together. Protein concentrations were 10 µM for both Hfq-WT and Hfq-NTR_65_, and 440 µM for the Hfq-CTR_11_ peptide. Protein concentrations were chosen in order to have similar mass concentrations.

5 µL of samples were then deposited on air plasma-cleaned EM grids (Lacey carbon films on 300 mesh copper grids). The excess was blotted with a filter paper and the grid was immediately plunged into a liquid ethane bath cooled with liquid nitrogen. Images were recorded on a Gatan Ultrascan camera using a 2200FS transmission electron microscope from JEOL Ltd operated at 200 kV. High magnification images were taken over large sections at random locations, in order to avoid statistical bias. Tomographic series were taken from regions of interest covering an angular range from −60° to + 60°. Statistics on membrane thickness were calculated from n = 20 pictures.

Images were processed with ImageJ software^54^ and tomogram reconstructions were calculated by the OS-SART method using the TomoJ plugin^55^. Stitching and quantitative analysis of the images were also carried out in ImageJ.

### Synchrotron Radiation Circular Dichroism (SRCD)

Measurements and data collection were carried out on DISCO beam-line at the SOLEIL Synchrotron (Gif-sur-Yvette, France)^56^. 0.25 mM (UV-absorption) Hfq-CTR_38_ was mixed with 1 mM of EPE or DOPG SUVs in 10 mM sodium phosphate, 20 mM NaCl, pH 7.5. After 20 min incubation, 2–4 µL of samples were loaded into circular demountable CaF_2_ cells of 30 microns’ path length^57^. Three separated data collections with fresh sample preparations were carried out to ensure consistency and repeatability. Spectral acquisitions of 1 nm steps at 1.2 integration time, between 260 and 180 nm were performed in triplicate for the samples as well as for the baselines (consisting of buffer alone or buffer + SUVs). (+)-camphor-10-sulfonic acid (CSA) was used to calibrate amplitudes and wavelength positions of the SRCD experiment. Data-analyses including averaging, baseline subtraction, smoothing, scaling and standardization were carried out with CDtool^58^. Secondary structure content was determined using BestSel^59^. Normalized root-mean-square deviation (NRMSD) indicated the most accurate fit for each spectrum; values of <0.15 were considered significant.

### Data availability

All data generated or analyzed during this study are included within this article and the Supplementary information or are available from the corresponding author upon request.

## Electronic supplementary material

Sup Figures and table
