# Publisher Correction: Membrane association of the bacterial riboregulator Hfq and functional perspectives

**DOI:** 10.1038/s41598-017-15019-y

**Published:** 2017-11-10

**Authors:** Antoine Malabirade, Javier Morgado-Brajones, Sylvain Trépout, Frank Wien, Ileana Marquez, Jérôme Seguin, Sergio Marco, Marisela Velez, Véronique Arluison

**Affiliations:** 1 Laboratoire Léon Brillouin LLB, CEA, CNRS UMR12, Université Paris Saclay, CEA Saclay, 91191 Gif-sur-Yvette, France; 2 Instituto de Catálisis y Petroleoquímica, CSIC, c/ Marie Curie, 2, Cantoblanco, E-28049 Madrid, Spain; 3 Institut Curie, Research Center, PSL Research University, Chemistry, Modelisation and Imaging for Biology (CMIB) Bât 110-112, Centre Universitaire, 91405 Orsay, France; 4 INSERM U 1196, CNRS UMR 9187, Université Paris Saclay, Université Paris-Sud, Bât 110-112, Centre Universitaire, Rue Henri Becquerel, 91405 Orsay, France; 5 DISCO Beamline, Synchrotron SOLEIL, 91192 Gif-sur-Yvette, France; 6 Institute for Integrative Biology of the Cell (I2BC), CEA, CNRS, Univ. Paris-Sud, Université Paris-Saclay, 91198 Gif-sur-Yvette, Cedex France; 7 Université Paris Diderot, 75013 Paris, France

*Scientific Reports* **7**:10724; doi:10.1038/s41598-017-11157-5; Article published online 06 September 2017

The original version of this Article contained an error in the name of the author Véronique Arluison, which was incorrectly given as Arluison Véronique. This has now been corrected in the PDF and HTML versions of the Article.

