# Supplementary material for: Membrane association of the bacterial riboregulator Hfq and functional perspectives
Source: Sci Rep. 2017 Sep 6;7:10724. doi: 10.1038/s41598-017-11157-5 (PMC5587644; doi:10.1038/s41598-017-11157-5)
Supplement: Supplementary file 1 — Sup Figures and table [file 41598_2017_11157_MOESM1_ESM.pdf]

Supplementary material

Membrane association of the bacterial riboregulator Hfq and functional perspectives

Antoine Malabirade, Javier Morgado-Brajones, Sylvain Trépout, Frank Wien, Ileana Marquez, Jérôme Seguin, Sergio Marco, Marisela Velez and Véronique Arluison

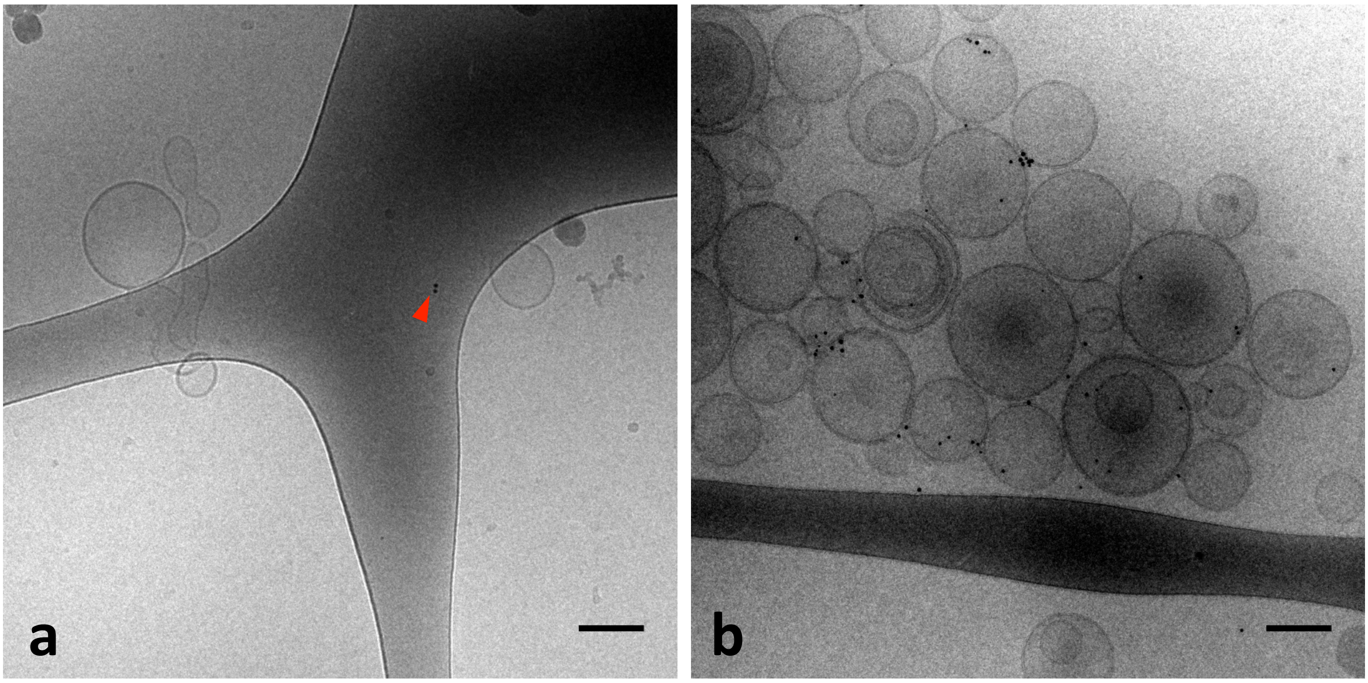

Fig. S1: ***a.*** EPE SUVs and 4nM NiNTA-NanoGold beads. The few beads found do not bind lipidic membranes and prefer to stick to the carbon film (red arrow). ***b.*** EPE SUVs incubated with wild-type Hfq and 4nM NiNTA-NanoGold beads. The four Histidines naturally present in the C-terminal tails of Hfq are sufficient to bind some beads, indicating that His-tag labelling does not significantly influences the protein-membrane interaction. Scale bars 100 nm.

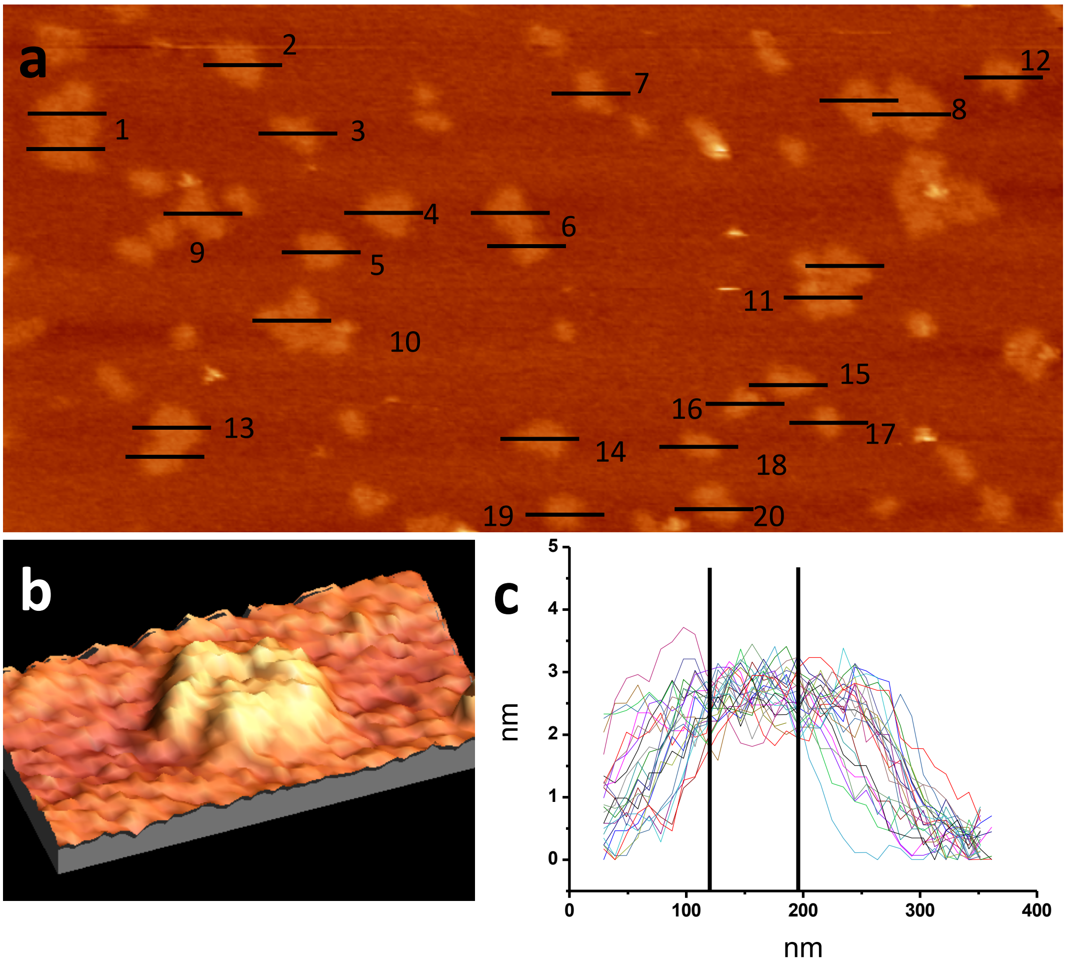

Fig. S2: Height measurement of Hfq aggregates on an EPE lipid bilayer by liquid AFM. a. Aggregates and profiles used to obtain the average height. **b.** The 3D representation of one aggregate shows that the surface is not flat. **c.** Compilation of all the profiles used. We selected the heighest profile of each aggregate, which is shown as a black line in panel a, and the plateau region that was selected to average is indicated between two lines in panel c. The average height measured from 20 aggregates is 2.7 ± 0.2 nm.

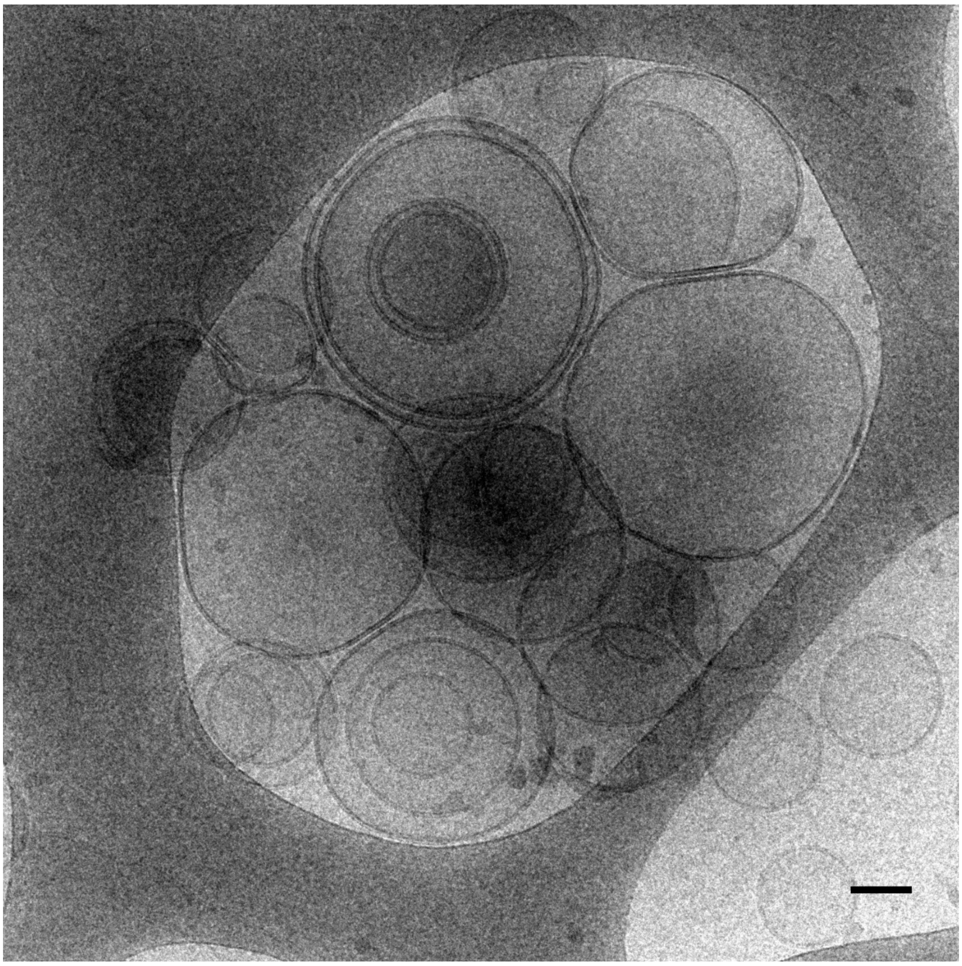

Fig. S3: Experiment with Hfq-NTR$_{65}$. Cryo-TEM image showing that the truncated torus is able to bind and regroup some liposomes but without visible effect on the membrane thickness. Scale bar: 50 nm.

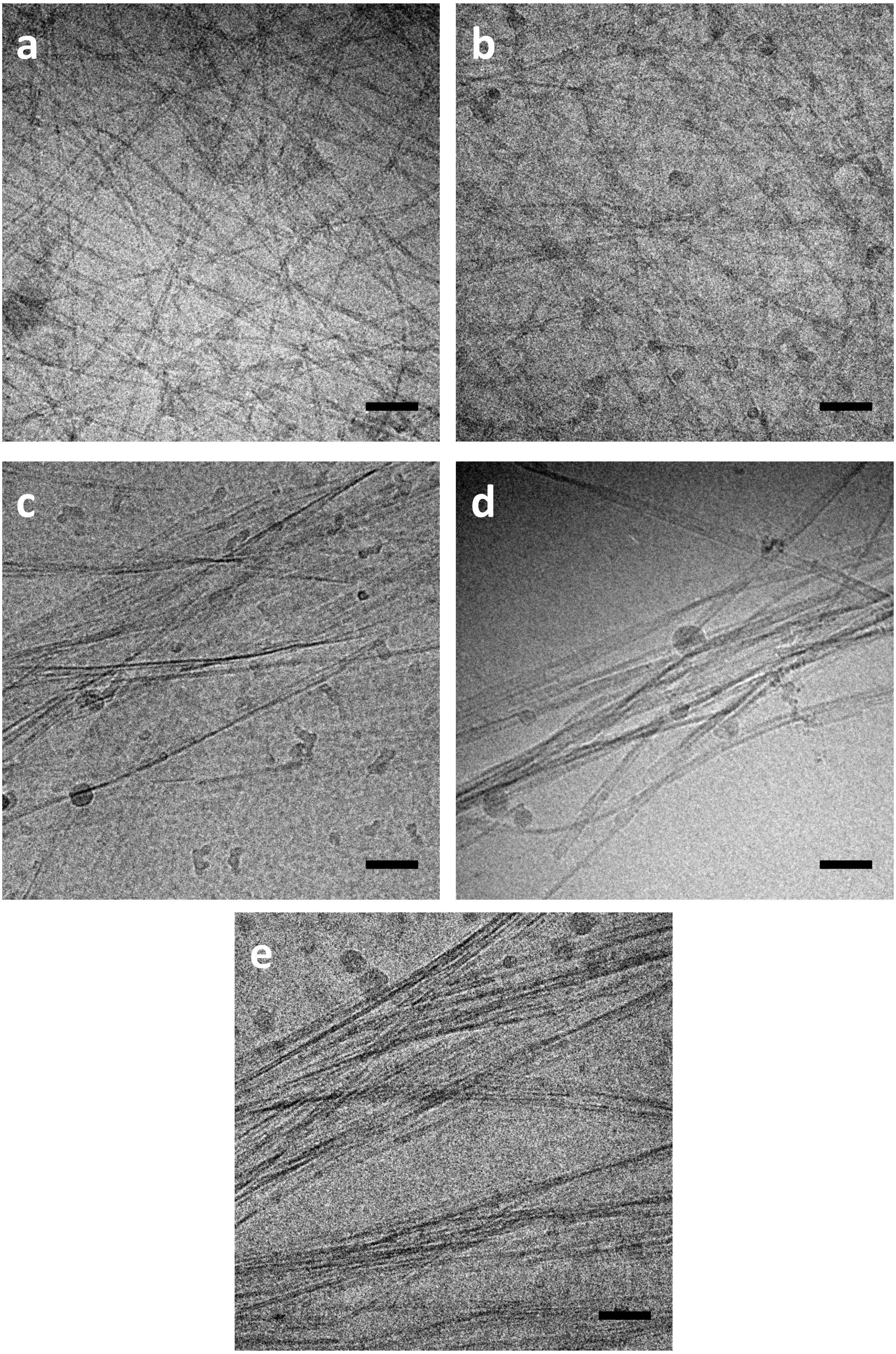

Fig. S4: Hfq-CTR peptides successfully tested for amyloid fibers formation by Cryo-TEM. ***a-e***: Hfq-CTR_{38}, Hfq-CTR_{26}, Hfq-CTR_{17a}, Hfq-CTR_{17b}, Hfq-CTR_{11} respectively. Concentration 0.5 g.L^{-1}, scale bar 50 nm.

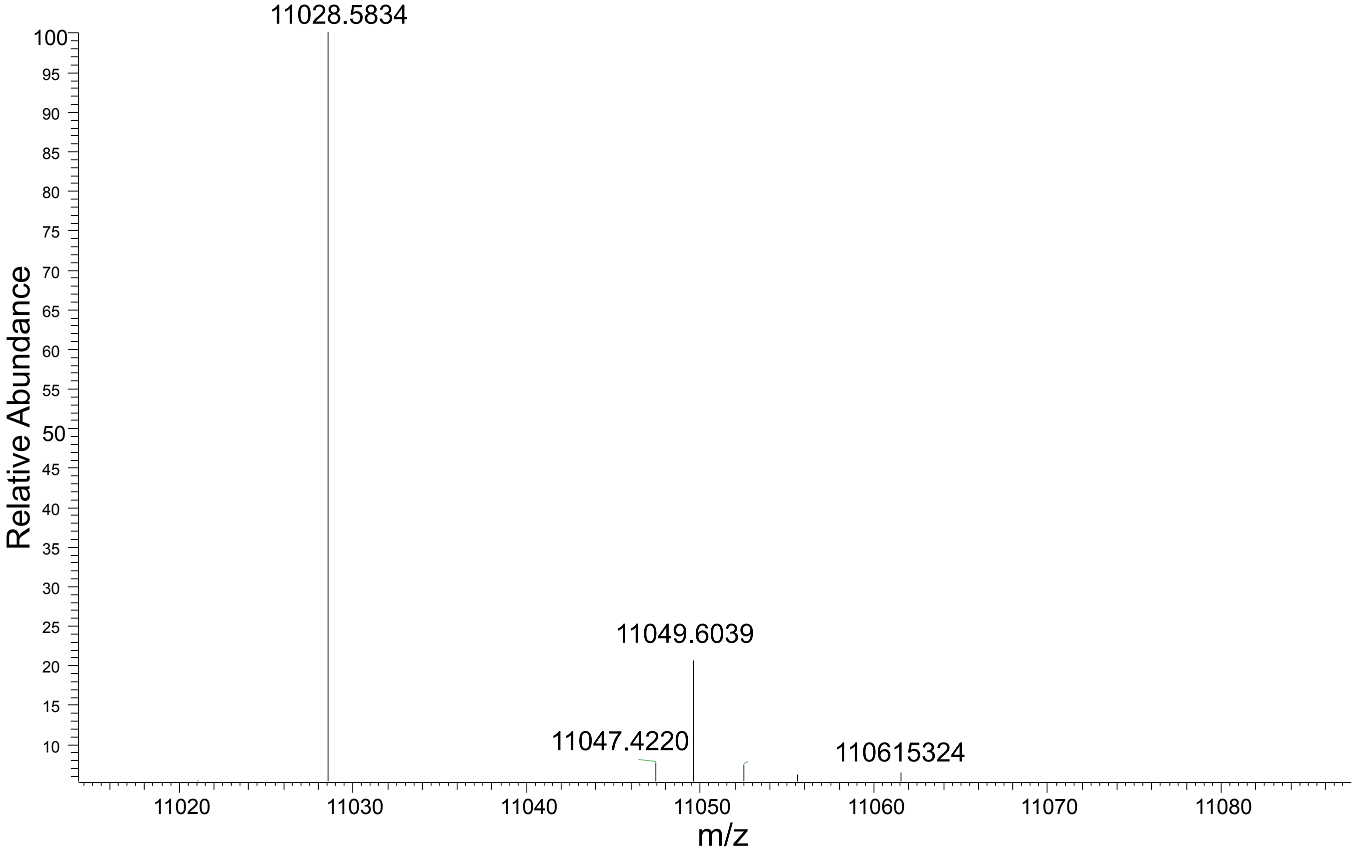

Fig. S5: Mono-isotopic deconvolued spectra of ESI/Orbitrap Mass-Spectrometry on purified native Hfq. The majoritary pic at 11028.58 Da corresponds to a monomer of native Hfq (in accordance with Expasy Compute pI/Mw: 11 028,54 Da). The 11049.60 Da pic probably corresponds to Hfq+Na^+. The protein solution was exchanged on a C4-reverse phase Ziptip (Merck Millipore) with 60% acetonitrile in water before electrospray.

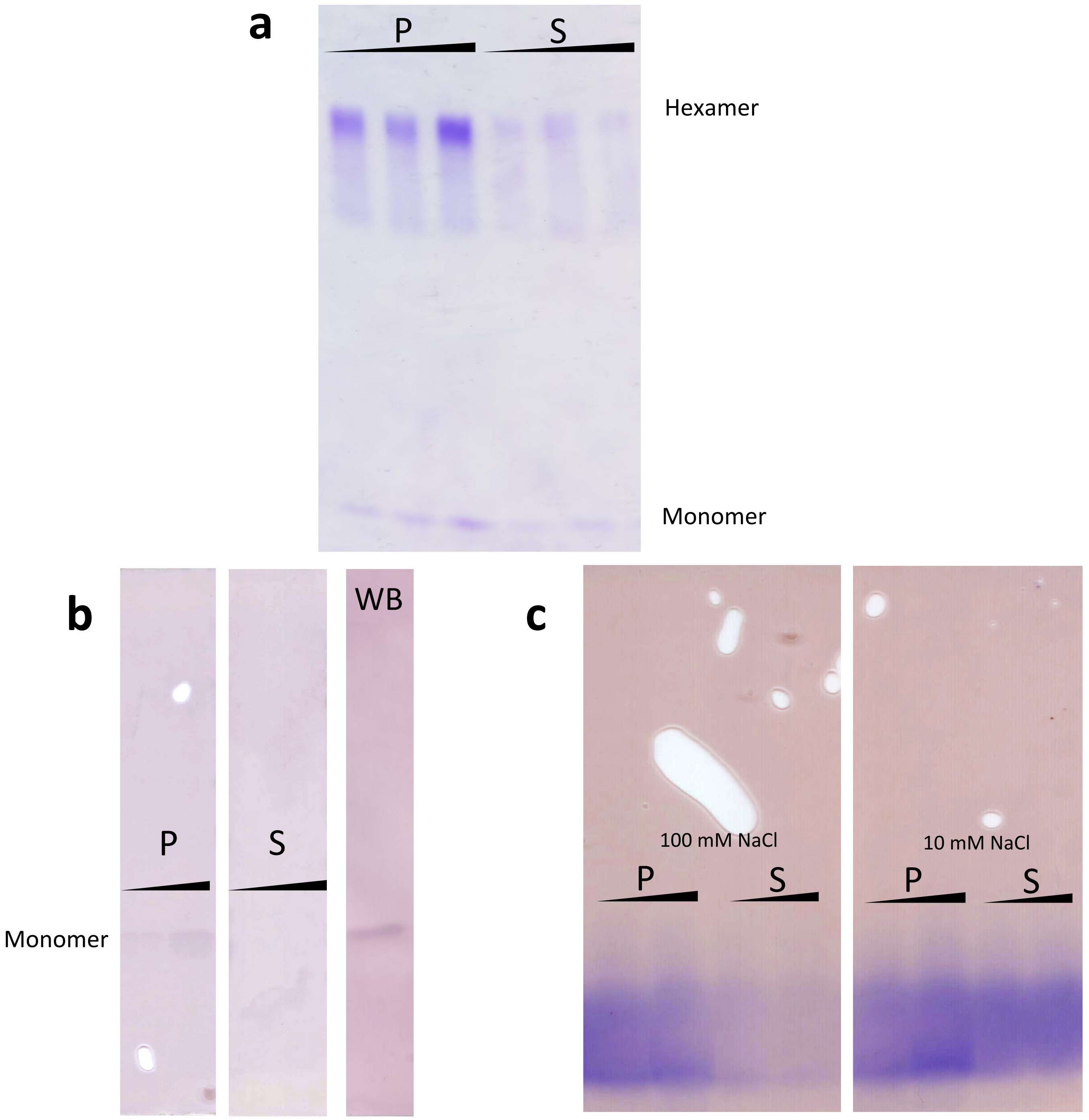

Fig. S6: Full SDS-PAGE for Hfq co-sedimentation assay (EPE). ***a.*** Wild-type Hfq with increasing concentrations of EPE (100, 300, 600µM); gel percentage 8-16%. **b**. Hfq-NTR_{65} (EPE 300, 600µM); gel percentage 4-20%. **c.** Hfq-CTR38 peptides (EPE 300, 600µM) in the presence of 100 and 10 mM NaCl; gel percentage 12%. P, Pellet fraction; S, Supernatant fraction. As previously described, WT Hfq hexamers are only partially denatured by SDS-PAGE and migrate mostly as hexamers, while truncated Hfq-NTR_{65} is less stable and can be dissociated on the PAGE[25,60,61]. In ***b***, a Western Blot (WB) is also provided to confirm the nature of Hfq-NTR_{65} (rat anti-Hfq IgG).

Sup Table S1: Hfq CTR peptides tested for amyloidogenic assembly

Region	Amino acid sequence	Amyloid fibers
Hfq Wild Type	MAKGQSLQDPFLNALRRERVPVSIYLVNGIKLQGQIESFD QFVILLKNTVSQMVYKHAISTVVPSRPVSHHSNNAGGGT SSNYHHGSSAQNTSAQQDSEETE	
Hfq-CTR_{38}	SRPVSHHSNNAGGGTSSNYHHGSSAQNTSAQQDSEETE	**Yes**
-	SRPVSHHSNNAGGGT	No
Hfq-CTR_{26}	GGTSSNYHHGSSAQNTSAQQDSEETE	**Yes**
-	VSHHSNNAGGGTSSNYHHGS	No
-	GTSSNYHHGSSAQNTSA	No
Hfq-CTR_{17a}	GSSAQNTSAQQDSEETE	**Yes**
Hfq-CTR_{17b}	NYHHGSSAQNTSAQQDS	**Yes**
-	NTSAQQDSEETE	No
-	SAQQDSEETE	No
Hfq-CTR_{11}	SAQNTSAQQDS	**Yes**
